# Supplementary material for: Association of Pulmonary Function with Osteosarcopenic Obesity in Older Adults Aged over 50 Years
Source: Nutrients. 2023 Jun 28;15(13):2933. doi: 10.3390/nu15132933 (PMC10346328; doi:10.3390/nu15132933)
Supplement: Supplementary file 1 [file nutrients-15-02933-s001.zip › nutrients-2462179-supplementary.pdf]

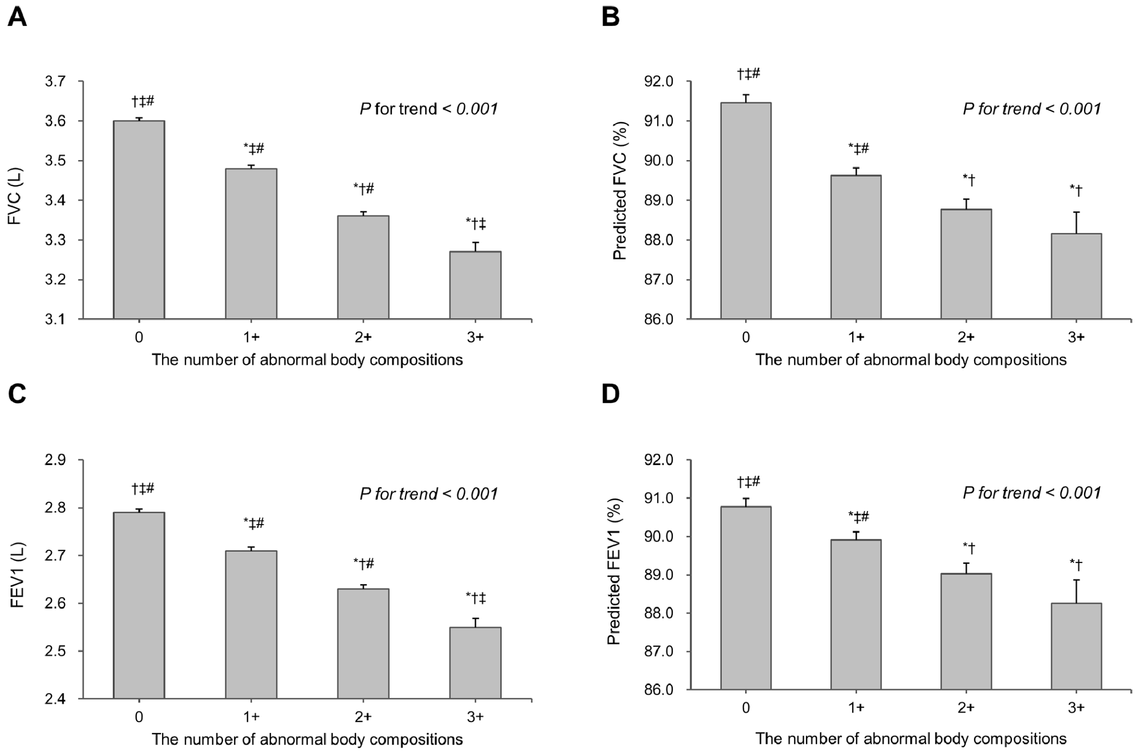

Supplementary Figure S1. Pulmonary Function Mean Values (SE) According to Abnormal Body Compositions.

*Adjusted p<0.05 compared with the normal group in post hoc analysis.

†Adjusted p<0.05 compared with the one component group in post hoc analysis.

‡Adjusted p<0.05 compared with the two component group in post hoc analysis.

#Adjusted p<0.05 compared with the OSO group in post hoc analysis.
